# Determinants of the number of dental visits in the general adult population in Germany during the COVID-19 pandemic

**DOI:** 10.1186/s12913-025-12577-0

**Published:** 2025-03-22

**Authors:** André Hajek, Hans-Helmut König, Berit Lieske, Loujain Wees, Tjore Model, Larissa Zwar, Ghazal Aarabi

**Affiliations:** 1 Department of Health Economics and Health Services Research, University Medical Center Hamburg-Eppendorf, Hamburg Center for Health Economics, Martinistraße 52, 20246 Hamburg, Germany; 2 Department of Periodontics, Preventive and Restorative Dentistry, Center for Dental and Oral Medicine, University Medical Center Hamburg-Eppendorf, Martinistraße 52, 20246 Hamburg, Germany

**Keywords:** Dental service use, Andersen model, Dental care use, Dental service utilization, Dental attendance

## Abstract

**Background:**

Oral health is essential to general health and well-being. The utilization of oral health care services represents an important factor in reducing oral health morbidities. In order to understand the disparities in the frequency of dental visits, it is necessary to identify determinants that influence the use of those services. The aim of the current study was to investigate the determinants of the number of dental visits in Germany during the COVID-19 pandemic.

**Methods:**

We used data from the general adult population in Germany with *n* = 2,807 individuals in the analytical sample. Average age was 46.5 years (SD: 15.2 years, range 18 to 74 years) and 48.2% of the individuals were female. The number of dental visits in the preceding 12 months served as outcome measure. Grounded on the extended Andersen model, various determinants were included in regression analysis. Multiple negative binomial regressions were used.

**Results:**

Negative binomial regressions showed that a higher number of dental visits was significantly associated with personality-related (higher conscientiousness, IRR: 1.09, 95% CI: 1.03–1.15; higher neuroticism, IRR: 1.06, 95% CI: 1.00-1.12) and psychosocial factors (higher loneliness, IRR: 1.12, 95% CI: 1.02–1.22). In contrast, only very few predisposing characteristics, and none of the enabling resources and need factors were significantly associated with the outcome measure.

**Conclusions:**

This study particularly emphasized the importance of personality-related factors and psychosocial factors (in terms of loneliness) for the number of dental visits during the pandemic. These factors, often overlooked in prior research, deserve further attention in upcoming studies dealing with the number of dental visits.

**Supplementary Information:**

The online version contains supplementary material available at 10.1186/s12913-025-12577-0.

## Background

Oral diseases are among the most common chronic health conditions, affecting over 3.5 billion people globally [1]. Over the last 30 years, the number of untreated oral conditions has steadily increased by 50%, making it a major global health concern [2]. Especially, since neglecting oral health can have serious consequences on one’s mental [3, 4] and social well-being [5, 6] as well as quality of life [7]. Moreover, poor oral health demonstrates a risk factor for other chronic diseases, such as diabetes or cardiovascular disease [8, 9]. An important part of oral health care and a prophylactic factor in reducing the risk of oral morbidities is the utilization of dental care services. Studies show that routine dental visits are associated with better oral health outcomes [10, 11]. Thus, in order to promote and maintain good oral health, the provision of adequate oral health care access is essential.

In 2011, the overall mean number of dental visits for community-dwelling individuals ≥ 40 years in Germany was 2.1 [12]. The insured person-related utilization rates of dental services in Germany are above average with around 70% when compared to other countries globally [13]. According to the 5th German Oral Health Study (DMS V), reported dental visits within the last 12 months were 84% for the 35–44 age group and as high as 90% for the younger senior citizens (65–74 years) [14]. However, when looking at social, demographic, regional, and general health aspects, we see significant disparities with regards to the utilization of dental services within populations [15, 16].

Research conducted on barriers to dental care utilization and dental treatment avoidance showed associations with overall health status [17, 18], age [12], gender [19], and education level as well as socio-economic status [20, 21]. Furthermore, dental visiting behaviour is influenced by individual psychological and personality related factors [22–24]. For example, studies demonstrate that conscientiousness as a personality trait is associated with the use of preventive dental visits and adherence to treatment [23, 25, 26]. In order to understand the disparities of the number of visits between individuals, it is necessary to identify the determinants that influence the use of those services.

The COVID-19 pandemic had widespread effects on healthcare systems, challenged dental health professionals, and has negatively impacted the oral health status of populations [27]. One noticeable impact has been on the frequency of dental visits. According to data from a nationally representative online-survey in Germany (COSMO Study), more than a fifth of the respondents reported postponed dental visits during the pandemic [28]. In addition, the pandemic-related restrictions such as social distancing and lockdowns influenced the economic performance of dental practices in Germany [29]. These measures further added to a decrease in dental consultations, particularly in the field of prevention, periodontics and prosthodontics [30, 31].

In light of the unmet oral health target and the impact of COVID-19 on dental service utilization, there is a pressing need to explore additional determinants of dental visit frequency. Our aim was to investigate those determinants– comprehensively and broadly - within the extended Andersen behavioral model of healthcare utilization. This model allows for more comprehensive understanding of health services utilization behaviours, as it categorizes factors into predisposing characteristics (e.g., gender or age), enabling resources (e.g. income), need factors (e.g. chronic illnesses, self-rated health), psychosocial factors (e.g. loneliness), and personality-related factors (e.g. conscientiousness or neuroticism) [32].

## Methods

### Sample

We used data from a quota-based online survey of individuals aged 18 to 74 years who lived in Germany. The data collection took place in spring 2022 (i.e., March 2022). An established market research company called Bilendi and respondi were responsible for the recruitment of the individuals– which is a certified company (ISO 26362).

Participants were selected via an online sample with the aim of ensuring that the distribution of age, sex and state reflected the distribution of these parameters in the general adult population in Germany. In total, 11,900 individuals were invited to participate in the current study. We were not able to calculate a potential selection bias because we did not have the data available. In sum, 3,091 participants took part in this study. Due to a few missings, our analytical sample equalled *n* = 2,807 observations.

Informed consent was provided by all individuals. Study approval was given by the Psychological Ethics Committee of the University Medical Center Hamburg-Eppendorf (LPEK-0412).

### Dependent variable: number of dental visits

Individuals were first asked to report whether or not (i.e., no or yes) they had a visit at the dentist in the past twelve months. Individuals should also count home visits as well. In contrast, picking up a prescription did not count as dental visit. If individuals replied with “yes”, they should report the actual number of dental visits in the past twelve months. Based on these two questions, a continuous variable was generated (from 0 upwards) to quantify the number of dental visits.

### Determinants

A wide array of determinants were chosen (and entered simultaneously) for regression analysis based on previous research [33] following the extended Andersen’s model [34, 35] distinguishing between predisposing characteristics, enabling resources, need factors, psychosocial factors and personality-related factors. More precisely, we particularly refer to the idea of Hajek and König [35] who argued very vigorously in favour of greater consideration of psychosocial and personality-related factors in the Andersen model.

With regard to predisposing characteristics, we included age, sex (men; women; diverse), marital status (widowed; divorced; single; living separately: married or in partnership; living together: married or in a partnership), education at school level (upper secondary school; qualification for applied upper secondary school; polytechnic secondary school; intermediate secondary school; currently in school training/education; without school-leaving qualification/lower secondary school), having a migration background (no; yes), and labour force participation (full-time employed; retired; other).

With regard to enabling resources, we focused on income. To this end, we used the monthly household net income (the categories were as follows: under EUR 500, 500 EUR to lower than EUR 1000, EUR 1000 to lower than EUR 1500, EUR 1500 to lower than EUR 2000, EUR 2000 to lower than EUR 2500, EUR 2500 to lower than EUR 3000, EUR 3000 to lower than EUR 3500, EUR 3500 to lower than EUR 4000, EUR 4000 to lower than EUR 4500, EUR 4500 to lower than EUR 5000, EUR 5000 to lower than EUR 6000, EUR 6000 to lower than EUR 8000, EUR 8000 or more; not reported). We used a median-split in regression analysis.

With regard to need factors, we included: having at least one chronic disease (no; yes), self-rated health (1 = “very poor”, 2 = “poor”, 3 = “average”, 4 = “good”, and 5 = “very good”), depressive symptoms, anxiety symptoms, and being vaccinated against COVID-19 (no; yes). To quantify depressive symptoms, we used the Patient Health Questionnaire-9 (PHQ-9) [36] which ranges from 0 to 27 whereby higher values reflect more depressive symptoms. To quantify anxiety symptoms, we used the Generalized Anxiety Disorder-7 (GAD-7) [37] which ranges from 0 to 21 whereby higher values reflect more anxiety symptoms. Furthermore, lifestyle factors reflect need factors and can be important for dental service use [33]. We included these lifestyle factors: smoking status (from “yes, daily” to “never smoker”), alcohol consumption (from “never” to “daily”), and sports activities (from “no sports activity” to “more than 4 hours a week”).

With regard to personality-related factors, we included the 10-item Big Five Inventory (BFI-10) [38] to measure personality. More precisely, this tool quantifies agreeableness, conscientiousness, extraversion, neuroticism and openness to experience (each with two dimensions, thus the BFI-10 tool has ten items in total). Each dimension ranges from 1 to 7, whereby higher values indicate a more pronounced personality factor.

With regard to psychosocial factors, we included: perceived social isolation, loneliness and coronavirus anxiety. Perceived social isolation was assessed using the 4-item Bude and Lantermann tool [39]. This tool ranges from 1 to 4, whereby higher values correspond to a higher level of perceived social isolation. To measure loneliness, the 6-item De Jong Gierveld loneliness tool [40] was used– which ranges from 1 to 4, with higher values reflecting higher levels of loneliness. The Coronavirus Anxiety Scale (CAS) [41–43] was used to measure the coronavirus anxiety consisting of five items. The sum score ranges from 0 to 20, with higher values reflecting higher levels of coronavirus anxiety.

### Statistical analysis

First, sample characteristics were displayed. Subsequently, a negative binomial regression model was calculated– which is a common way when dealing with the frequency of dental visits as outcome [44] (further model specifications are shown in Supplementary Table 1). A negative binomial regression model, for instance, had lower Akaike Information Criterion (AIC) and Bayesian Information Criterion (BIC) values when compared to a Poisson model (Poisson model, AIC: 9,238.42, BIC: 9,476.02; negative binomial model, AIC: 8,847.59, BIC: 9,091.13). This demonstrates that the negative binomial model fits our data better compared to the Poisson model. Variance inflation factors (VIFs) were calculated. However, they were rather low (e.g., mean VIF of 1.6, highest VIF of 3.8) indicating that multicollinearity is not a serious threat.

The significance level was set at *p* < 0.05. Stata 16.1 (Stata Corp., College Station, Texas) was used to perform statistical analyses.

## Results

### Sample characteristics

Sample characteristics for our total analytical sample are presented in Table 1. The average age was 46.5 years (SD: 15.2 years, range 18 to 74 years) and 48.2% of the individuals were female. The average number of dental visits in the preceding 12 months equaled 1.4 (SD: 1.8; 28.7% reported no dental visits). Further details are shown in Table 1.

**Table 1:** Sample characteristics for the analytical sample (*n* = 2,807)

Variables	Mean (SD) / *n* (%)
Gender	
Male	1448 (51.6)
Female	1353 (48.2)
Diverse	6 (0.2)
Age	46.5 (15.2)
Marital status	
Single/Divorced/Widowed/Living separately: Married or in partnership	1129 (40.2)
Living together: Married or in a partnership	1678 (59.8)
Education	
Upper secondary school	1114 (39.7)
Qualification for applied upper secondary school	315 (11.2)
Polytechnic Secondary School	185 (6.6)
Intermediate Secondary School	873 (31.1)
Lower Secondary School	304 (10.8)
Currently in school training/education	11 (0.4)
Without school-leaving qualification	5 (0.2)
Migration background	
No	2465 (87.8)
Yes	342 (12.2)
Household net income	
Lower than median	1249 (44.5)
Higher than median	1558 (55.5)
Employment status	
Full-time employed	1263 (45.0)
Retired	593 (21.1)
Other	951 (33.9)
Smoking status	
Yes, daily	671 (23.9)
Yes, sometimes	218 (7.8)
No, not anymore	864 (30.8)
Never smoker	1054 (37.5)
Alcohol consumption	
Daily	186 (6.6)
Several times a week	515 (18.3)
Once a week	424 (15.1)
1–3 times a month	486 (17.3)
Less often	669 (23.8)
Never	527 (18.8)
Sports activities	
No sports activity	759 (27.0)
Less than one hour a week	523 (18.6)
Regularly, 1–2 h a week	698 (24.9)
Regularly, 2–4 h a week	449 (16.0)
Regularly, more than 4 h a week	378 (13.5)
Chronic diseases	
Absence of at least one chronic disease	1506 (53.7)
Presence of at least one chronic disease	1301 (46.3)
Self-rated health (from 1 = very bad to 5 = very good)	3.6 (0.9)
Being vaccinated against COVID-19	
No	318 (11.3)
Yes	2489 (88.7)
Depressive symptoms (PHQ-9, 0 to 27, higher values reflect more depressive symptoms)	6.2 (5.4)
Anxiety symptoms (GAD-7, 0 to 21, higher values reflect more anxiety symptoms)	5.0 (4.7)
Extraversion (BFI-10, from 1 to 7, higher values reflect higher extraversion)	4.0 (1.2)
Agreeableness (BFI-10, from 1 to 7, higher values reflect higher agreeableness)	5.2 (1.1)
Conscientiousness (BFI-10, from 1 to 7, higher values reflect higher conscientiousness)	5.6 (1.1)
Neuroticism (BFI-10, from 1 to 7, higher values reflect higher neuroticism)	3.1 (1.3)
Openness to experience (BFI-10, from 1 to 7, higher values reflect higher openness)	5.0 (1.1)
Coronavirus anxiety (CAS, from 0 to 20, higher values reflect higher coronavirus anxiety)	1.5 (3.1)
Perceived social isolation (Bude Lantermann Scale, 1 to 4, higher values reflect higher perceived social isolation)	1.9 (0.8)
Loneliness (De Jong Gierveld Loneliness Scale, 1 to 4, higher values reflect higher loneliness)	2.1 (0.7)
Number of dental visits (0 upwards)	1.4 (1.8)

### Regression analysis

Results of multiple negative binomial regression analysis are displayed in Table 2. A higher number of dental visits was significantly associated with personality related factors– in terms of a higher level of conscientiousness (IRR: 1.09, 95% CI: 1.03–1.15) and a higher level of neuroticism (IRR: 1.06, 95% CI: 1.00–1.12). Furthermore, a higher number of dental visits was significantly associated with psychosocial factors– in terms of a higher level of loneliness (IRR: 1.12, 95% CI: 1.02–1.22).

**Table 2:** Determinants of number of dental visits. Results of multiple negative binomial regression analysis

**Independent variables**	**Number of dental visits**
Sex: - Female (Ref.: Men)	1.08 (0.96 - 1.22)
- Diverse	1.51+ (0.98 - 2.33)
Age (in years)	1.00 (1.00 - 1.01)
Marital status: - Living together (married or in a partnership) (Ref.: widowed; divorced; single; living separately: married or in partnership)	1.04 (0.93 - 1.18)
Highest educational degree: - Qualification for Applied Upper Secondary School (Ref.: Upper Secondary School)	0.95 (0.83 - 1.10)
- Polytechnic Secondary School	1.00 (0.85 - 1.19)
- Intermediate Secondary School	0.98 (0.87 - 1.10)
- Lower Secondary School	0.98 (0.78 - 1.22)
- Currently in school training/education	3.72* (1.16 - 11.96)
- Without school-leaving qualification	0.29+ (0.07 - 1.27)
Migration background: - Yes (Ref.: No)	1.03 (0.85 - 1.25)
Household net income group: - Above median (Ref.: Below median)	1.03 (0.89 - 1.19)
Employment status: - Retired (Ref.: Full-time employed)	0.99 (0.87 - 1.14)
- Other	0.88* (0.79 - 0.98)
Smoking status: - Yes, daily (Ref.: Never smoker)	0.96 (0.83 - 1.10)
- Yes, sometimes	0.90 (0.75 - 1.07)
- No, not anymore	1.01 (0.90 - 1.13)
Alcohol consumption: - Daily (Ref.: Never)	0.91 (0.75 - 1.12)
- Several times a week	0.96 (0.83 - 1.11)
- Once a week	0.98 (0.84 - 1.13)
- 1–3 times a month	0.98 (0.85 - 1.13)
- Less often	1.04 (0.89 - 1.21)
Sports activities: - Less than one hour a week (Ref.: No sports activity)	0.93 (0.81 - 1.07)
- Regularly, 1–2 hours a week	1.11 (0.96 - 1.28)
- Regularly, 2–4 hours a week	1.03 (0.89 - 1.18)
- Regularly, more than 4 hours a week	1.04 (0.90 - 1.20)
Chronic diseases: - Presence of at least one chronic disease (Ref.: Absence of chronic diseases)	1.07 (0.97 - 1.18)
Self-rated health	1.01 (0.95 - 1.07)
Being vaccinated against COVID-19: - Yes (Ref.: No)	1.05 (0.84 - 1.31)
Depressive symptoms	1.01 (1.00 - 1.03)
Anxiety symptoms	1.00 (0.98 - 1.01)
Extraversion	1.04+ (1.00 - 1.08)
Agreeableness	0.97 (0.92 - 1.01)
Conscientiousness	1.09** (1.03 - 1.15)
Neuroticism	1.06* (1.00 - 1.12)
Openness to experience	1.00 (0.95 - 1.05)
Coronavirus anxiety	1.00 (0.98 - 1.01)
Perceived social isolation	0.92+ (0.84 - 1.01)
Loneliness	1.12* (1.02 - 1.22)
Intercept	0.49 (0.27 - 0.90)
Observations	2,807
Pseudo R^2^	0.01

Incidence rate ratios are reported; robust standard errors in parentheses; *** *p *<0.001, ** *p *<0.01, * *p *<0.05, + *p *<0.10

In contrast, very few predisposing characteristics and none of the enabling resources (in terms of income) and need factors were significantly associated with the number of dental visits in the preceding 12 months– except for currently in school training/education (compared to upper secondary school, IRR: 3.72, 95% CI: 1.16–11.96) and employment situation (other compared to full-time employed, IRR: 0.88, 95% CI: 0.79–0.98). Worth noting that different model specifications are presented in Supplementary Table 1. It should be noted that the effect sizes were consistently small in our regression model following existing recommendations [45].

## Discussion

### Key findings

Based on data from a large survey of the general adult population, we aimed to explore the determinants of the number of dental visits during the COVID-19 pandemic. Our study showed that a higher number of dental visits was significantly associated with personality-related (higher conscientiousness and higher neuroticism) and psychosocial factors (higher loneliness). On the contrary, only very few predisposing characteristics, and none of the enabling resources and need factors were significantly associated with the outcome measure.

### Personality-related factors

Our results align with previous findings regarding the role of conscientiousness in dental visit frequency. Individuals with higher conscientiousness tend to engage in more positive health behavior and preventive actions overall [25, 46–49], including more regular dental visits [23, 26].

With regard to neuroticism, while a number of previous studies demonstrated that neuroticism or neuroticism-related characteristics are linked to fewer dental visits and dental visit procrastination [23, 26, 50–52], we, on the other hand, found a significant association between neuroticism and a higher number of dental visits. Similarly, in a previously published systematic review on personality-related factors and healthcare use, it was found that higher neuroticism was associated with increased utilization of healthcare services overall [53]. The tendency for increased care seeking behavior was also reported in other studies [54, 55]. One important theoretical position is that people with higher levels of neuroticism engage in poorer health behaviors, such as smoking, a common risk factor for poorer oral health outcomes [56, 57]. Furthermore, neurotic people are more likely to experience heightened anxiety levels [58, 59]. In consequence, neuroticism could lead to poorer health via anxiety-provoked maladaptive behaviors. This, in turn, could lead to necessary dental visits. Another possible explanation is that neuroticism as a personality trait is associated with excessive worrying and poor coping mechanisms [60], especially during extreme situations like the COVID-19 pandemic [61]. In response, we hypothesize that higher neuroticism might lead to higher levels in worry or fear of disease, thus leading to more frequent visits. It should also be noted that the majority of studies with findings opposite to ours were conducted before the pandemic or COVID-19-related determinants were not specifically scrutinized. From another perspective than personality theory, it could be important to consider that large-scale health crises, such as the COVID-19 pandemic, represent a unique and extreme situation that might evoke psychological maladjustments or negative affect. For example, according to a German large-scale experience-sampling study by Kroencke et al. [62], their study subjects high in neuroticism experienced more negative affect in daily life during the COVID-19 pandemic. In turn, higher negative affect has been associated with frequent dental visits in a large German sample of non-institutionalized individuals [63], which might explain the differing findings. Hence, the complexity of individuals’ psychological and healthcare responses during extreme health-related events such as pandemics calls for further investigation on the role of personality traits in order to fully understand and address these disparities in dental care-seeking behaviors.

### Psychosocial factors

Our results revealed an increased number of dental visits among those with higher loneliness. Similarly, other research reported positive associations between loneliness and making physician visits prior to the pandemic [64, 65]. Among a sample of 5,514 rural elderly people in China, the researchers observed an association between loneliness and higher odds of utilizing physician visits, after controlling for the effects of other covariates [66]. One probable explanation for our findings lies in the previously documented association between poorer oral health and higher loneliness [67]. This could suggest that those individuals with higher levels of loneliness might have poorer oral health status, which could have prompted unavoidable dental visits during the pandemic.

Nonetheless, the evidence regarding the role of loneliness is mixed. In contrast to our study results, Burr et al. [68] found an opposite association between perceived social isolation, measured with a loneliness index, and lower probability to seek dental care. The study employed data from the 2008 Health and Retirement Study, a large, nationally representative survey among older persons in the United States. Yet, as social loneliness can partially be explained by social isolation, we speculate that there is most likely a number of pathways through which social relationships influence utilization patterns. Another important point to note is that loneliness levels and prevalence rates increased relative to pre-pandemic times [69] and presented a significant issue during the pandemic [70, 71]. This was highly influenced by COVID-19-related uncertainties and social distancing guidelines. Considering the severity of the situation, as previously mentioned, the effects of loneliness on dental service utilization might need to be interpreted differently depending on the situation.

### Predisposing characteristics, enabling resources and need factors

In our study, the differences in dental visit frequency between individuals with different predisposing characteristics, enabling factors and need factors were not pronounced. While age, sex, coronavirus anxiety as well as depression and anxiety symptoms were not significantly associated with the number of dental visits in the preceding 12 months, other studies demonstrated associations between these factors and dental visit avoidance during the pandemic [17, 28, 72]. The only two predisposing characteristics that showed a significant association with the number of dental visits were employment situation (other compared to full-time employed) and enrollment in school training/education at the time of data collection. The latter is somewhat surprising, since higher educational attainment has been identified to be associated with better dental service attendance [73–75]. Those individuals with ‘other’ employment status were significantly associated with fewer dental visits compared to full-time employed individuals. Those results are in line with other research, which found that people with better economic status visited dental clinics more frequently during the pandemic [27, 30, 76].

Overall, the heterogeneous results could be attributed to the uncertainty surrounding the pandemic, which led to precautionary measures including lockdowns, compulsory masks, vaccination recommendations/obligations, social distancing guidelines, and concerns about virus transmission in health care settings [31]. As a result, people with different enabling resources have been equally hesitant to seek dental care [31], thereby reducing the typical disparities observed before the pandemic. A number of studies from various countries comparing the frequency of dental visits before and during the COVID-19 pandemic have consistently shown a significant decrease in dental visits, along with increased hesitation in seeking dental care [30, 77, 78].

### Strengths and limitations

While there are some studies addressing the frequency of dental visits in Germany, to the best of our knowledge, this is the first study to investigate the determinants of the number of dental visits during COVID-19 using the extended Andersen Model to select determinants. Data for this study were obtained from a quota-based sample (age group, state, and sex) of the general adult population aged 18 to 74 years in Germany, ensuring representativeness. All determinants were quantified using established tools, and the effect sizes were consistently small in the regression model following existing recommendations. While a small recall bias may be present in the outcome measure, the use of a 12-month recall period aligns with existing recommendations [79].

However, it should be noted that the possibility of a selection bias cannot be dismissed. Additionally, the response rate for the survey was 26%, meaning a non-response bias cannot be excluded. Another important consideration is that our study did not distinguish between curative and preventive dental visits, which may represent a crucial need factor, an important determinant of dental service use. Individuals who visit the dentist only when experiencing pain or discomfort are considered problem-oriented visitors, focusing solely on addressing acute dental problems. Such problem-oriented visits may initially drive the use of dental services. This, in turn, could lead to changes in perceived health status. Without accounting for dental need characteristics and differentiating between reasons for dental visits, it becomes challenging to predict dental health behavior patterns or the distribution of oral health problems. Therefore, we recommend that future research should incorporate both self-perceived (perceived need) and actual oral health status (evaluated need) as well as the reasons for dental visits [33]. Moreover, it is important to highlight that while regularity of dental attendance (e.g., regular dental check-ups, only when having symptoms, only in extreme pain, never) has been commonly used to describe dental attendance behavior, the unique context of the COVID-19 pandemic required additional considerations. COVID-19 forced the implementation of precautionary measures, leading to disruptions in dental check-up routines. In many cases, dental practitioners had to reschedule, postpone or combine checkups and minor treatments in a single visit to minimize patient exposure. This external factor influenced the patterns of dental attendance [28, 80, 81].

Furthermore, our study only included the sole presence of at least one chronic condition (no or yes) in regression analysis. However, some chronic conditions (e.g., diabetes or certain autoimmune diseases) may be of greater importance for dental visits than other chronic conditions (e.g., allergic conditions). Thus, we strongly recommend future research focusing on the association between specific chronic conditions and the number of dental visits. Moreover, according to the Andersen model, a perceived need may also reflect other factors not captured in our current study. In addition, the study subjects were not asked whether they had a permanent dentist, which was found to influence delayed dental visit behavior in a positive direction in the study by Hoshino et al. [26]. Lastly, our study has a cross sectional design, which limits our ability to establish causality. Further studies using longitudinal data would provide more conclusive insights and highlight more complex processes.

## Conclusions

After adjusting for various factors, this study particularly emphasized the importance of personality-related factors and psychosocial factors (in terms of loneliness) for the number of dental visits. These factors are often overlooked in prior research and deserve further attention in upcoming studies dealing with dental care utilization. Integrating personality traits and psychosocial factors into patient-centered oral health planning, alongside socioeconomic and demographic considerations, holds significant potential for improving access to and adherence to dental care. Tailoring health interventions to specific personality traits and employing health literacy strategies designed for different personality types could further enhance patient engagement and health-related behaviors [82, 83].

In light of the new challenges brought by the COVID-19 pandemic, we must be careful to what extent certain determinants are affected by COVID-related impacts. We clearly recommend future research in the post-pandemic era. It remains to be seen which factors are significantly associated with the number of dental visits in this new era. Moreover, future research could focus on moderating effects (e.g., education x loneliness).

## Supplementary Information

Supplementary Material 1. Table 1

## Data Availability

Data are not publicly available due to ethical restrictions, but interested parties may contact the authors for more information.

## Abbreviations

AIC: Akaike Information Criterion
BFI-10: 10-item Big Five Inventory
BIC: Bayesian Information Criterion
CAS: Coronavirus Anxiety Scale
GAD-7: Generalized Anxiety Disorder-7
PHQ-9: Patient Health Questionnaire-9
