# Supplementary material for: Determinants of the number of dental visits in the general adult population in Germany during the COVID-19 pandemic
Source: BMC Health Serv Res. 2025 Mar 22;25:420. doi: 10.1186/s12913-025-12577-0 (PMC11929202; doi:10.1186/s12913-025-12577-0)
Supplement: Supplementary file 1 — Supplementary Material 1. Table 1 [file 12913_2025_12577_MOESM1_ESM.docx]

**Table 1 Determinants of number of dental visits. Results of multiple negative binomial regressions.**

| **Independent variables** | **Number of dental visits** | **Number of dental visits** | **Number of dental visits** | **Number of dental visits** |
| --- | --- | --- | --- | --- |
|  |  |  |  |  |
| Sex: - Female (Ref.: Men) | 1.14* | 1.12* | 1.11+ | 1.08 |
|  | (1.03 - 1.26) | (1.00 - 1.26) | (0.99 - 1.24) | (0.96 - 1.22) |
| - Diverse | 1.60* | 1.55* | 1.57+ | 1.51+ |
|  | (1.06 - 2.41) | (1.01 - 2.38) | (1.00 - 2.46) | (0.98 - 2.33) |
| Age (in years) | 1.00 | 1.00 | 1.00+ | 1.00 |
|  | (1.00 - 1.01) | (1.00 - 1.01) | (1.00 - 1.01) | (1.00 - 1.01) |
| Marital status: - Living together (married or in a partnership) (Ref.: widowed; divorced; single; living separately: married or in partnership) | 1.02 | 1.02 | 1.03 | 1.04 |
|  | (0.90 - 1.16) | (0.90 - 1.16) | (0.91 - 1.18) | (0.93 - 1.18) |
| Highest educational degree: - Qualification for Applied Upper Secondary School (Ref.: Upper Secondary School) | 0.95 | 0.95 | 0.95 | 0.95 |
|  | (0.82 - 1.09) | (0.82 - 1.10) | (0.82 - 1.09) | (0.83 - 1.10) |
| - Polytechnic Secondary School | 1.00 | 1.00 | 0.99 | 1.00 |
|  | (0.85 - 1.19) | (0.85 - 1.18) | (0.83 - 1.17) | (0.85 - 1.19) |
| - Intermediate Secondary School | 0.98 | 0.99 | 0.98 | 0.98 |
|  | (0.87 - 1.10) | (0.88 - 1.10) | (0.87 - 1.10) | (0.87 - 1.10) |
| - Lower Secondary School | 0.97 | 0.97 | 0.96 | 0.98 |
|  | (0.77 - 1.23) | (0.77 - 1.22) | (0.77 - 1.20) | (0.78 - 1.22) |
| - Currently in school training/education | 3.46* | 3.72* | 3.70* | 3.72* |
|  | (1.09 - 11.04) | (1.11 - 12.43) | (1.09 - 12.62) | (1.16 - 11.96) |
| - Without school-leaving qualification | 0.28 | 0.28 | 0.29 | 0.29+ |
|  | (0.05 - 1.60) | (0.05 - 1.54) | (0.06 - 1.50) | (0.07 - 1.27) |
| Migration background: - Yes (Ref.: No) | 1.05 | 1.05 | 1.03 | 1.03 |
|  | (0.85 - 1.31) | (0.86 - 1.29) | (0.84 - 1.27) | (0.85 - 1.25) |
| Household net income group: - Above median (Ref.: Below median) | 1.03 | 1.03 | 1.04 | 1.03 |
|  | (0.89 - 1.20) | (0.88 - 1.19) | (0.89 - 1.20) | (0.89 - 1.19) |
| Employment status: - Retired (Ref.: Full-time employed) | 1.01 | 1.01 | 0.98 | 0.99 |
|  | (0.88 - 1.17) | (0.88 - 1.16) | (0.86 - 1.12) | (0.87 - 1.14) |
| - Other | 0.89* | 0.88* | 0.87* | 0.88* |
|  | (0.79 - 0.99) | (0.79 - 0.99) | (0.78 - 0.98) | (0.79 - 0.98) |
| Smoking status: - Yes, daily (Ref.: Never smoker) |  | 0.97 | 0.96 | 0.96 |
|  |  | (0.85 - 1.11) | (0.83 - 1.10) | (0.83 - 1.10) |
| - Yes, sometimes |  | 0.90 | 0.88 | 0.90 |
|  |  | (0.75 - 1.07) | (0.73 - 1.06) | (0.75 - 1.07) |
| - No, not anymore |  | 1.03 | 1.02 | 1.01 |
|  |  | (0.92 - 1.15) | (0.91 - 1.14) | (0.90 - 1.13) |
| Alcohol consumption: - Daily (Ref.: Never) |  | 0.94 | 0.91 | 0.91 |
|  |  | (0.76 - 1.15) | (0.75 - 1.12) | (0.75 - 1.12) |
| - Several times a week |  | 0.97 | 0.97 | 0.96 |
|  |  | (0.84 - 1.12) | (0.84 - 1.12) | (0.83 - 1.11) |
| - Once a week |  | 0.98 | 0.98 | 0.98 |
|  |  | (0.84 - 1.14) | (0.85 - 1.14) | (0.84 - 1.13) |
| - 1-3 times a month |  | 0.99 | 0.99 | 0.98 |
|  |  | (0.85 - 1.14) | (0.86 - 1.14) | (0.85 - 1.13) |
| - Less often |  | 1.05 | 1.04 | 1.04 |
|  |  | (0.89 - 1.23) | (0.89 - 1.23) | (0.89 - 1.21) |
| Sports activities: - Less than one hour a week (Ref.: No sports activity) |  | 0.92 | 0.93 | 0.93 |
|  |  | (0.80 - 1.06) | (0.81 - 1.07) | (0.81 - 1.07) |
| - Regularly, 1-2 hours a week |  | 1.09 | 1.10 | 1.11 |
|  |  | (0.94 - 1.27) | (0.95 - 1.28) | (0.96 - 1.28) |
| - Regularly, 2-4 hours a week |  | 1.01 | 1.03 | 1.03 |
|  |  | (0.87 - 1.16) | (0.89 - 1.19) | (0.89 - 1.18) |
| - Regularly, more than 4 hours a week |  | 1.03 | 1.06 | 1.04 |
|  |  | (0.89 - 1.19) | (0.92 - 1.22) | (0.90 - 1.20) |
| Chronic diseases: - Presence of at least one chronic disease (Ref.: Absence of chronic diseases) |  |  | 1.08 | 1.07 |
|  |  |  | (0.97 - 1.19) | (0.97 - 1.18) |
| Self-rated health |  |  | 1.01 | 1.01 |
|  |  |  | (0.95 - 1.07) | (0.95 - 1.07) |
| Being vaccinated against COVID-19: - Yes (Ref.: No) |  |  | 1.04 | 1.05 |
|  |  |  | (0.81 - 1.33) | (0.84 - 1.31) |
| Depressive symptoms |  |  | 1.01 | 1.01 |
|  |  |  | (0.99 - 1.02) | (1.00 - 1.03) |
| Anxiety symptoms |  |  | 1.00 | 1.00 |
|  |  |  | (0.99 - 1.02) | (0.98 - 1.01) |
| Extraversion |  |  |  | 1.04+ |
|  |  |  |  | (1.00 - 1.08) |
| Agreeableness |  |  |  | 0.97 |
|  |  |  |  | (0.92 - 1.01) |
| Conscientiousness |  |  |  | 1.09** |
|  |  |  |  | (1.03 - 1.15) |
| Neuroticism |  |  |  | 1.06* |
|  |  |  |  | (1.00 - 1.12) |
| Openness to experience |  |  |  | 1.00 |
|  |  |  |  | (0.95 - 1.05) |
| Coronavirus anxiety |  |  |  | 1.00 |
|  |  |  |  | (0.98 - 1.01) |
| Perceived social isolation |  |  |  | 0.92+ |
|  |  |  |  | (0.84 - 1.01) |
| Loneliness |  |  |  | 1.12* |
|  |  |  |  | (1.02 - 1.22) |
| Intercept | 1.11 | 1.11 | 0.91 | 0.49 |
|  | (0.87 - 1.40) | (0.80 - 1.54) | (0.57 - 1.46) | (0.27 - 0.90) |
|  |  |  |  |  |
| Observations | 2,807 | 2,807 | 2,807 | 2,807 |
|  |  |  |  |  |
| Pseudo R² | 0.01 | 0.01 | 0.01 | 0.01 |

Incidence rate ratios are reported; robust standard errors in parentheses; *** p<0.001, ** p<0.01, * p<0.05, + p<0.10. In the first model specification, socioeconomic determinants were included; in the second model specification, lifestyle-factors were additionally included; in the third model specification, health-related factors were also included; in the final model, psychosocial and personality-related factors were also included.
